# Application and clinical efficacy of comprehensive nursing intervention in pelvic floor and bladder function recovery post-cervical cancer surgery

**DOI:** 10.12669/pjms.40.10.9271

**Published:** 2024-11

**Authors:** Qiang Zhao

**Affiliations:** Qiang Zhao, Department of Gynaecology and Obstetrics, Tangshan People’s Hospital, Tangshan 063000, Hebei, China

**Keywords:** Comprehensive nursing intervention, Post-cervical cancer surgery, Pelvic floor function, Bladder function

## Abstract

**Objective:**

To evaluate the clinical efficacy of comprehensive nursing intervention in pelvic floor and bladder function recovery post-cervical cancer surgery.

**Method:**

This was a retrospective study. A total of 120 patients with cervical cancer undergoing radical surgery at Tangshan People’s Hospital from June 2021 to June 2023 were randomly divided into two groups (n=60 cases each group). The control group received routine nursing intervention, while the study group was provided with comprehensive nursing intervention. Comparative analysis was conducted on clinical indicators, postoperative complications, bladder and pelvic floor function recovery, and patient satisfaction before and after intervention in both groups.

**Result:**

In the study group, ambulation time, gastrointestinal function recovery, duration of postoperative drainage tube placement, oral diet resumption time, duration of postoperative vaginal bleeding, and length of hospital stay were significantly shorter than in the control group (all *p*< 0.05). Bladder function recovery in the study group was superior to that in the control group (*p*< 0.05). The study group showed lower scores for anorectal disorders, urogenital disorders, and pelvic organ prolapse than the control group (*p*< 0.05, respectively). Patient satisfaction was 100% in the study group and 85% in the control group, suggesting a statistically significant difference between the two groups (*p*< 0.05).

**Conclusion:**

Comprehensive nursing intervention deserve to be promoted as it can accelerate postoperative clinical recovery, reduce the incidence of surgical complications, improve pelvic floor and bladder function post-cervical cancer surgery, and enhance patient satisfaction with care.

## INTRODUCTION

In China, cervical cancer is one of the most commonly occurring malignant tumors in women, second only to breast cancer.^1^,^2^ Statistics^3^ show that there are 530,000 new cases of invasive cervical cancer every year, leading to erosion, ulcers, and bleeding in the vaginal and perineal mucosal tissues, causing pain, fear, and unbearable living conditions for patients. It has a high incidence and mortality rate, severely affecting the life and health of the female population.^4^,^5^ Surgery is currently the most effective treatment for cervical cancer, with hysterectomy combined with pelvic lymph node dissection being a common approach. However, due to the extensive surgical scope, various factors such as physical function limitations, nerve damage, and long-term indwelling catheters postoperatively can induce multi-organ dysfunction, including bladder and pelvic floor dysfunction, with an incidence ranging from 12% to 53% and clinical manifestations like urinary and storage dysfunction or abnormalities, pelvic organ prolapse, or constipation.^6^,^7^ Considering that no specific and effective treatment is available for bladder and pelvic floor dysfunction after radical surgery for cervical cancer, early postoperative rehabilitation guidance and prospective nursing play a positive role in improving prognosis.^8^

Early scientific rehabilitation training is crucial for improving postoperative bladder function in cervical cancer. However, relying on routine nursing makes it challenging to provide systematic postoperative recovery guidance for bladder function in patients with cervical cancer. There is a lack of objectivity in determining postoperative correct activities for patients. The comprehensive nursing intervention theory aims to promote the overall health of patients in combination with social cognitive theory, self-regulation theory, and behavior promotion theory. It identifies individual nursing needs and issues, provides targeted nursing decisions, and helps individuals improve and maintain health behaviors, thus achieving an ideal health status.^9^ This theory has been widely applied in chronic disease management. Therefore, the current study applied the comprehensive nursing intervention model to clinical nursing for post-cervical cancer surgery patients, to evaluate the clinical efficacy of comprehensive nursing intervention in pelvic floor and bladder function recovery post-cervical cancer surgery.

## METHODS

This was a retrospective study. One-hundred and twenty patients who were confirmed to have cervical cancer and underwent radical surgery at Tangshan People’s Hospital from June 2021 to June 2023 were divided into control group(n=60) and study group(n=60) according to different nursing intervention methods. We have obtained the above sample size according to the actual situation of the hospital and statistical estimation. No significant differences were observed between the two groups in general patient data, indicating a high level of comparability (Table-I).

**Table-I:** Comparative analysis of general patient data between the study and control groups (*χ̅*±*s*), n = 60.

Item	Study group	Control group	t/χ^2^	p
Age (years)	46.37±10.45	47.72±9.83	0.73	0.47
Tumor diameter (cm)	3.27±1.08	3.35±1.13	0.40	0.69
Clinical staging			0.41	0.52
Stage I (%)	16 (27%)	13 (22%)		
Stage II (%)	44 (73%)	47 (78%)		
Pathological type			0.54	0.46
Squamous-cell carcinoma (%)	26 (43%)	30 (50%)		
Adenocarcinoma (%)	18 (30%)	17 (28%)		
Adenosquamous carcinoma (%)	16 (27%)	13 (22%)		
Marital status			0.15	0.70
Unmarried	12 (20%)	10 (17%)		
Married	41 (68%)	39 (65%)		
Divorced	7 (12%)	11 (18%)		

p > 0.05.

### Ethical Approval:

The study was approved by the Institutional Ethics Committee of Tangshan People’s Hospital (No.: 2020019; date: October 15, 2020), and written informed consent was obtained from all participants.

### Inclusion criteria:

- Confirmed diagnosis of cervical cancer through histopathology and pelvic MRI^10^;
- Proficiency in listening, speaking, reading, and writing;
- Radical surgery for cervical cancer;
- Age 25~70 years;
- Ability for self-care (Karnofsky Performance Score ≥ 80)^11^;
- Complete clinical data;
- Willingness to cooperate with the study and good treatment compliance.

### Exclusion criteria:

- Concurrent malignancies in other parts of the body;
- Severe organ dysfunction;
- Coagulation and endocrine disorders;
- Metastatic cervical cancer;
- Cognitive or behavioral abnormalities hindering study completion;
- Preoperative radiotherapy or chemotherapy;
- Stubborn urinary system infections that cannot be satisfactorily corrected;
- Preexisting bladder functional disorders such as diabetes, neuropathy, and spinal cord injuries.

### Treatment methods:

The control group underwent the standard perioperative intervention, including the following procedures: Upon admission, routine health education was conducted, and preoperative examinations were completed. Vital signs of patients were monitored, and postoperative dietary instructions included advice to minimize the consumption of fried, smoked, high-fat, and high-protein food, while encouraging the intake of food rich in carbohydrates and dietary fiber. Preoperative education was provided by urology nurses, focusing on surgical preparation. Postoperatively, patients received care related to daily activities, diet, and medication. This involved timely replacement of the urinary bag to maintain catheter patency, daily hygiene of the external genitalia, and ensuring the patient’s daily water intake exceeded 2000 ml. Starting from one to two weeks postoperatively, intermittent catheterization was initiated, and the timing for catheter removal was determined based on the residual bladder urine volume of the patient.

The study group adopted a comprehensive rehabilitation intervention model, building upon the intervention applied in the control group. The key components were outlined as follows:

- Collection of patient data and comprehensive assessment upon admission, correcting patients’ unhealthy habits related to physical activity and diet.
- Tailoring individualized, staged goals and self-management plans based on the patient’s condition and individual circumstances; goals aimed at improving pelvic floor and bladder functions and enhancing overall quality of life.
- Explaining the definition, risks, causes, and importance of rehabilitation exercises for pelvic floor and bladder dysfunction after cervical cancer surgery, and reflecting disease-related behaviors, daily life behaviors, and self-concept behaviors, covering topics such as cervical cancer overview, diagnosis, treatment, daily life issues, personalized dietary recommendations, and the importance of pelvic floor and bladder rehabilitation training
- Instructing patients in
- Guidance on bladder function exercises: i) timed and prompted voiding exercises: scheduling specific times for voiding based on the patient’s urination pattern; ii) delayed voiding exercises: instructing patients to learn to control the frequency of urination, gradually extending daytime intervals from two hours to four hours and adjusting nighttime urination to 2–3 times under self-awareness control; iii) mindful voiding exercises: five minutes before urination or catheterization, guiding patients to lie down and maintain full-body relaxation, and encouraging them to imagine being in a quiet bathroom, listening to the sound of flowing water, and helping them mentally prepare for urination, followed by slow emptying of the bladder by nursing staff or gradual catheterization; iv) compensatory voiding exercises: for patients with insufficient activity in the sphincter and detrusor muscles, using breath-holding techniques to assist in emptying the bladder, and instructing patients to sit, lean forward, hold their breath, exert abdominal pressure, and perform a forceful bowel movement-like action.
- Facilitating group discussions to explore coping strategies, such as discussing the benefits of personal health behaviors and emphasizing the importance of home exercises.
- Maintenance actions: i) established chat groups for continuous disease management post-discharge, regularly sharing disease-related management knowledge within the group; instructed patients to maintain detailed daily journals based on their conditions, uploaded weekly updates in picture format to the group; and sent phone reminders to those who fail to upload promptly, ensured continuous reinforcement of patient health beliefs, behaviors, and self-management abilities.

### Outcome measures:

### Clinical indicators:

ambulation time, gastrointestinal recovery time, drainage tube indwelling time, time from surgery to the first meal, duration of postoperative vaginal bleeding, and length of hospital stay between the two groups.

### Postoperative complications:

cervical adhesions, wound bleeding, abnormal menstruation, and infection.

### Bladder function:

On the day of catheter removal, patients were encouraged to drink a large amount of water and void voluntarily. Residual urine volume was measured using ultrasound, and bladder function was graded on the following scale 12: i) Grade I (normal): residual urine volume < 50 ml, representing normal bladder function; ii) Grade II (good): residual urine volume 50–100 ml, denoting good bladder function recovery; iii) Grade III (fair): residual urine volume > 100 ml, indicating fair bladder function recovery; iv) Grade IV (poor): difficulty in urination.

Comparative analysis of pelvic floor function: Evaluation using the Pelvic Floor Disability Index (PFDI-20)^13^, comprising 20 items organized into three subscales: anorectal disorders, urogenital disorders, and pelvic organ prolapse. Each item is scored on a 0–4 scale, with a total score ranging from 0 to 300. Higher scores indicate greater pelvic floor dysfunction.

### Patient satisfaction comparative analysis:

Utilizing the Patient Satisfaction Questionnaire Short Form (PSQ-18)^14^ for a comparative analysis of patient satisfaction before and after intervention. Levels of satisfaction were classified as “very satisfied”, “more than satisfied”, “satisfied”, “uncertain”, and “dissatisfied”. Overall satisfaction was calculated as (very satisfied + more than satisfied + satisfied) cases / total number of cases × 100%.

### Statistical analysis:

Statistical analysis was performed using SPSS 20.0. Measurement data were expressed as “mean ± standard deviation (*χ̅*±*S*)”. For intergroup data analysis, the independent samples t-test was employed, while intragroup data analysis utilized the paired t-test. The comparison of rates was conducted using the chi-square test. *P*< 0.05 was considered statistically significant.

## RESULTS

A comparative analysis of clinical outcomes between the two groups is presented in Table-II. The study group exhibited significantly faster ambulation time and gastrointestinal recovery, shorter drainage tube indwelling time, faster postoperative resumption of oral diet, longer duration of postoperative vaginal bleeding, and longer hospital stay compared to the control group (*p* = 0.00, respectively). The study group exhibited a postoperative complication rate of 8%, significantly lower than the control group with a complication rate of 23%. This difference was statistically significant (*p* = 0.02) (Table-III).

**Table-II:** Comparative analysis of clinical outcomes between the two groups (*χ̅*±*S*), n = 60.

Group	Ambulation time (d)	Gastrointestinal recovery time (d)	Drainage tube indwelling time (d)	Oral diet resumption time (d)	Duration of vaginal bleeding (d)	Postoperative length of hospital stay (d)
Study group	2.53±0.27	3.60±0.31	4.78±1.33	1.38±0.28	8.03±1.34	7.50±1.68
Control group	3.21±0.43	4.35±0.32	5.46±1.39	2.47±0.35	10.82±3.06	9.42±1.39
*t*	10.37	13.04	2.76	18.83	6.47	6.82
*p*	0.00	0.00	0.00	0.00	0.00	0.00

p > 0.05.

**Table-III:** Comparative analysis of postoperative complications between the two groups (*χ̅*±*S*), n = 60.

Group	Cervical adhesions	Wound bleeding	Abnormal menstruation	Infection	Incidence (%)
Study group	0	3	0	2	5 (8%)
Control group	4	2	5	3	14 (23%)
*χ* ^2^					5.07
*p*					0.02

p > 0.05.

The study group showed a significantly higher proportion of Grade-I bladder function than the control group (*p* = 0.03) and a significantly lower proportion of Grade-III compared to the control group (*p* = 0.02). No significant differences were observed in the proportions of Grade II and IV bladder function between the two groups (*p* > 0.05, respectively). This suggests that bladder function recovery in the study group was superior to that in the control group (Table-IV).

**Table-IV:** Comparative analysis of bladder function between the two groups (*χ̅*±*S*), n = 60.

Group	Grade I*	Grade II	Grade III*	Grade IV
Study group	33	20	5	2
Control group	21	18	14	7
*χ* ^2^	4.85	1.48	5.07	3.00
*p*	0.03	0.22	0.02	0.08

* p < 0.05.

Before intervention, there was no statistically significant difference in pelvic floor dysfunction scores between the two groups (*P*> 0.05). After the intervention, the study group exhibited significantly lower scores for anorectal disorders, urogenital disorders, and pelvic organ prolapse compared to the control group (*P* = 0.00, respectively) (Table-V). The satisfaction rate in the study group was 100%, significantly higher than the 85% satisfaction rate in the control group (*p* = 0.00) (Table-VI).

**Table-V:** Comparative analysis of pelvic floor function between the two groups (*χ̅*±*S*), n = 60.

Item		Study group	Control group	t	p
Anorectal disorders	Before intervention	27.59±2.47	27.46±2.38	0.29	0.77
	After intervention*	30.87±5.28	38.72±6.03	7.58	0.00
Urogenital disorders	Before intervention	34.51±3.68	34.83±3.70	0.47	0.63
	After intervention*	40.92±4.63	47.51±4.68	7.55	0.00
Pelvic organ prolapse	Before intervention	24.76±2.33	24.69±2.48	0.16	0.87
	After intervention*	26.77±3.08	35.24±3.53	14.00	0.00

* p < 0.05.

**Table-VI:** Comparative analysis of patient satisfaction between the two groups (*χ̅*±*S*), n = 60.

Group	Very satisfied	More than satisfied	Satisfied	Uncertain	Dissatisfied	Overall satisfaction*
Study group	52	7	1	0	0	60 (100%)
Control group	23	16	12	3	6	51 (85%)
*χ* ^2^						9.73
*p*						0.00

* p < 0.05.

## DISCUSSION

This study demonstrates that the study group significantly outperformed the control group in clinical indicators such as ambulation time, gastrointestinal function recovery, drainage tube indwelling time, oral diet resumption time, duration of postoperative vaginal bleeding, and length of hospital stay(all *p* < 0.05). Moreover, the study group exhibited a significantly lower incidence of postoperative complications compared to the control group(*p*< 0.05). All this can be attributed to the comprehensive nursing intervention model’s ability to formulate rational plans based on the specific postoperative conditions of patients, thereby enhancing their compliance. The patient-centered philosophy of the comprehensive nursing intervention model maximally addresses patient needs, providing personalized and targeted nursing services, which proves beneficial for the early recovery of postoperative gastrointestinal function. Additionally, early postoperative guidance on passive movement and gradual transition to independent ambulation facilitates a swift recovery for patients in a relatively short period. Coppell et al.^9^ proposed that comprehensive nursing intervention revolves around the patient’s needs, preferences, and lifestyle, offering a holistic approach to nursing services and guidance, which is highly favorable for postoperative recovery.

At present, conventional nursing models in large medical centers, aside from assisting with routine clinical treatment, typically provide only general health guidance to patients, lacking comprehensive assessments that consider various aspects of the patient’s physical, mental, and behavioral well-being. Consequently, the outcomes are less than satisfactory. Comprehensive nursing intervention has emerged as a novel nursing approach in recent years. This model tailors care to a specific disease while considering the patient’s individual circumstances and follows predetermined methods of nursing care.^15^,^16^ Not only does it emphasize the patient’s physical rehabilitation, but it also prioritizes psychological care and behavioral guidance in a more personalized manner.^17^

Comprehensive nursing intervention contributes to the promotion of postoperative bladder and pelvic floor function recovery in patients with cervical cancer. It reduces the duration of catheterization, effectively prevents adverse reactions such as urinary retention, and enhances patients’ quality of life. The current study confirms that the recovery of bladder function in the study group was superior to that in the control group (*p*< 0.05). Additionally, scores for anorectal disorders, urogenital disorders, and pelvic organ prolapse in the study group were lower than those in the control group (all *p*< 0.05). All this might be explained by the comprehensive nursing approach targeting factors affecting postoperative bladder function recovery in patients with cervical cancer. Personalized nursing plans, including water intake schedules, perineal care, acupressure massage, anal contraction training, and catheter closure training, stimulate the bladder nerve system effectively. This stimulation accelerates nerve repair, enhances central nervous control, promotes bladder function recovery, shortens catheterization duration, and reduces the incidence of adverse reactions.^18^ Pelvic floor muscle exercises are the preferred method for promoting postoperative pelvic floor function recovery. In this study, repetitive pelvic floor muscle contractions at different times and intensities contributed to the elevation of muscle tone and strength. This can enhance pelvic floor support tension, improve pelvic floor blood circulation, and, consequently, promote pelvic floor function recovery. Simultaneously, consciously and effectively contracting pelvic floor muscles can constrict the urethra, increase urethral pressure, and aid in the synchronous improvement of bladder function.^18^

The comprehensive nursing intervention model not only addresses patients’ disease symptoms and physiological care but also emphasizes psychological intervention. Through psychological care, this approach helps patients eliminate negative emotions, and correct misconceptions about cervical cancer, its treatment, and recovery, thereby reducing the impact of psychological stress on prognosis. This approach facilitates the implementation of various measures and further promotes the recovery of bladder function in patients.^19^ Additionally, it significantly improves patients’ medical experiences, increases disease awareness, thereby reducing the generation of negative emotions, and enhances trust and satisfaction with healthcare professionals.^19^ Chandratre et al.^19^ suggested that the comprehensive nursing intervention model can enhance the nurse-patient relationship, improve patients’ psychological and emotional satisfaction, and enhance their sense of security. Compared to traditional nursing models, Shi X et al.^20^ believes that the comprehensive nursing intervention model is more readily accepted by patients, making it a more practical and ideal nursing model. Our study also confirms a 100% satisfaction rate in the study group compared to 85% in the control group, with the study group significantly higher than the control group (*p* < 0.05).

### Limitations:

It includes a modest sample size and a short follow-up period. In future clinical work, we aim to expand the sample size, extend the follow-up period, continuously assess and improve the intervention, and further broaden the application scope. This will enable us to validate the feasibility and effectiveness of this study.

## CONCLUSIONS

In conclusion, compared to traditional nursing approaches, the comprehensive nursing intervention model is more professional and reliable. It can promote rapid postoperative recovery, reduce surgical complications, improve postoperative pelvic floor and bladder function in cervical cancer patients, and enhance patient satisfaction with nursing care.

### Authors’ Contributions:

**Qiang Zhao** carried out the studies, participated in its design, collecting data and performed the statistical analysis, and drafted the manuscript, and are responsible and accountable for the accuracy or integrity of the work, and read and approved the final manuscript.

## References

[1] Nasioudis D, Albright BB, Haggerty AF, Ko EM, Kim SH, Morgan MA (2021). Survival following minimally invasive radical hysterectomy for patients with cervical carcinoma and tumor size ≤2 cm. Am J Obstet Gynecol.

[2] Aydogmus H, Sen S, Aydogmus S (2019). Pathological discrepancy between colposcopic directed cervical biopsy and conisation results:A five years experience of a single center in Turkey. Pak J Med Sci.

[3] Saida T, Sakata A, Tanaka YO, Ochi H, Ishiguro T, Sakai M (2019). Clinical and MRI Characteristics of Uterine Cervical Adenocarcinoma:Its Variants and Mimics. Korean J Radiol.

[4] Li YX, Chen F, Shi JJ, Huang YL, Wang M (2023). Convolutional Neural Networks for Classifying Cervical Cancer Types Using Histological Images. J Digit Imaging.

[5] Sadia H, Shahwani IM, Bana KFM (2022). Risk factors of cervical cancer and role of primary healthcare providers regarding PAP smears counseling:Case control study. Pak J Med Sci.

[6] Beckmann MW, Stuebs FA, Vordermark D, Koch MC, Horn LC, Fehm T (2021). The Diagnosis, Treatment, and Aftercare of Cervical Carcinoma. Dtsch Arztebl Int.

[7] Stolnicu S, Hoang L, Zhou Q, Iasonos A, Terinte C, Pesci A (2023). Cervical Adenosquamous Carcinoma:Detailed Analysis of Morphology, Immunohistochemical Profile, and Outcome in 59 Cases. Int J Gynecol Pathol.

[8] Sheng J, Xiang Y, Shang L, He Q (2020). Molecular alterations and clinical relevance in cervical carcinoma and precursors (Review). Oncol Rep.

[9] Coppell KJ, Abel SL, Freer T, Gray A, Sharp K, Norton JK (2017). The effectiveness of a primary care nursing-led dietary intervention for prediabetes:a mixed methods pilot study. BMC Fam Pract.

[10] Johnson CA, James D, Marzan A, Armaos M (2019). Cervical Cancer:An Overview of Pathophysiology and Management. Semin Oncol Nurs.

[11] Barbetta C, Allgar V, Maddocks M, Ribeiro C, Wilcock A, Currow DC (2022). Australia-modified Karnofsky Performance Scale and physical activity in COPD and lung cancer:an exploratory pooled data analysis. BMJ Support Palliat Care.

[12] Wein AJ (2022). Voiding Function and Dysfunction, Bladder Physiology and Pharmacology, and Female Urology. J Urol.

[13] Spencer JE, Brown HW, Oliphant SS (2021). Health literacy and PFDI-20 and PFIQ-7 completion in urogynecology patients. Int Urogynecol J.

[14] Thayaparan AJ, Mahdi E (2013). The Patient Satisfaction Questionnaire Short Form (PSQ-18) as an adaptable, reliable, and validated tool for use in various settings. Med Educ Online.

[15] Shimada M, Tsuji K, Shigeta S, Nagai T, Watanabe Z, Tokunaga H (2022). Rethinking the significance of surgery for uterine cervical cancer. J Obstet Gynaecol Res.

[16] Eastwood J, Maitland-Scott I (2020). Patient Privacy and Integrated Care:The Multidisciplinary Health Care Team. Int J Integr Care.

[17] Kaul S, Gupta M, Bandyopadhyay D, Hajra A, Deedwania P, Roddy E (2021). Gout Pharmacotherapy in Cardiovascular Diseases:A Review of Utility and Outcomes. Am J Cardiovasc Drugs.

[18] Ozdemir Koken Z, Sezer RE, Tosun K (2019). Nursing Care of the Patient with Neurogenic Bladder After Kidney Transplantation:A Case Report. Transplant Proc.

[19] Chandratre P, Mallen C, Richardson J, Muller S, Hider S, Rome K (2018). Health-related quality of life in gout in primary care:Baseline findings from a cohort study. Semin Arthritis Rheum.

[20] Shi X, Ma L, Hao J, Yan W (2021). Regulatory effects of comprehensive psychological intervention on adverse emotions and immune status of
cervical cancer patients during the perioperative period. Am J Transl Res.

